# Primary pseudomyogenic hemangioendothelioma of the vulva: a rare location for a rare entity

**DOI:** 10.1186/s13000-019-0846-9

**Published:** 2019-06-26

**Authors:** Yue-Fang Sun, Jian Wang

**Affiliations:** 1 Department of Pathology, Shanghai Tenth People’s Hospital, Tenth People’s Hospital of Tongji University, 301 Yanchang Middle Road, Shanghai, 200072 China; 2 Department of Pathology, Fudan University Shanghai Cancer Center, Shanghai, 200032 China

**Keywords:** Vulva, Pseudomyogenic hemangioendothelioma, Immunohistochemistry

## Abstract

**Background:**

Pseudomyogenic hemangioendothelioma (PMHE) is a recently described vascular neoplasm which typically occurs in the lower extremities of young to middle-aged adults.

**Case presentation:**

We present here a unique case of PMHE arising primarily in the vulva of a 51-year-old woman who presented with a painful vulvar nodule. Clinically, it was thought as Bartholin gland cyst, vulvar hematoma or papilloma. On surgery, two nodules were found with one located in the superficial dermis and the other in the deep subcutis. Histologically, these two nodules showed similar features, composed of fascicles or sheets of plump spindled to epithelioid cells with eosinophilic cytoplasm. Given the morphological resemblance to a myogenic tumor, the lesion was initially diagnosed as a rhabdomyosarcoma by the referring pathologist. However, a comprehensive reevaluation of the submitted slides made us reconsider a PMHE, which was subsequently confirmed by immunohistochemical study.

**Conclusion:**

This case demonstrates that PMHE can also develop in the female external genitalia albeit extremely rare. This disease should be included in the differential diagnostic list of vulvar tumors with spindled to epithelioid morphology and cytokeratin-positive immunophenotypes.

## Background

Pseudomyogenic hemangioendothelioma (PMHE), also known as epithelioid sarcoma-like hemangioendothelioma [1], is a rare vascular neoplasm of biologically intermediate malignancy. This rare tumor type typically occurs in the lower extremities of young to middle-aged adults with a striking male predominance [2]. Less commonly, it may involve upper extremities, trunk, pelvis, and head and neck region [3, 4]. Multicentric synchronous presentation with involvement of different tissue planes in an anatomic region is a common feature of PMHE. Due to the lack of morphological evidence of vasoformation, a vascular neoplasm is hardly considered by pathologists especially for those who lack expertise in soft tissue tumors. On the contrary, because of the cytomorphological striking resemblance to rhabdomyoblasts, it is not uncommon to misdiagnose PMHE as a myogenic tumor. In this report, we present such a case of PMHE that arose in the vulva of a middle-age woman which was initially thought as a rhabdomyosarcoma. To the best of our knowledge, this is the first case of PMHE arising primarily in the female external genitalia.

## Case presentation

The patient was a 51-year-old woman (gravida 2, para 2) who felt itchiness in the right vulva for 3 months. She scratched at whiles and recently noted a small painful nodule in the right vulva. She went to a clinic for medical attention. She was given external medicine for alleviation but turned to be less effective. She was then admitted to a local hospital for further treatment. Gynecological examination revealed two pea-sized solid nodules with tenderness affecting the right labium majus. Clinically, they were suspected as Bartholin gland cysts, vulvar hematomas or papillomas. On surgery, one nodule was found to be located in the superficial dermis and the other in the deep subcutis, both measuring approximately 1 cm in maximum diameter. Considering benign lesion, marginal resection was performed. It was interpreted pathologically as a rhabdomyosarcoma, with proliferative fasciitis needed to be excluded. In view of potential further treatment, the pathological materials were sent to us for further confirmation. After the final diagnosis of PMHE was rendered, the patient was recommended to have a thorough radiological examination, including PET-CT. There was no neoplastic disease elsewhere. A three-month-follow-up showed no signs of local recurrence or metastatic disease.

Grossly, one specimen tagged “mass of right labium majus (epidermis)” consisted of a 1.5 × 1 × 1 cm fibroadipose tissue covered with a 1.5 × 1 cm elliptical skin. On cut section, there was a solid gray nodule, measuring 1.5 × 0.8 × 0.5 cm in size and was intermediate to firm in consistency. The other specimen tagged “mass of right labium majus (deep)” consisted mainly of adipose tissue, measuring 2 × 1 × 0.8 cm in total volume. On cut section, there was presence of solid grayish area, measuring about 1 cm in maximum size.

**Fig. 1:**
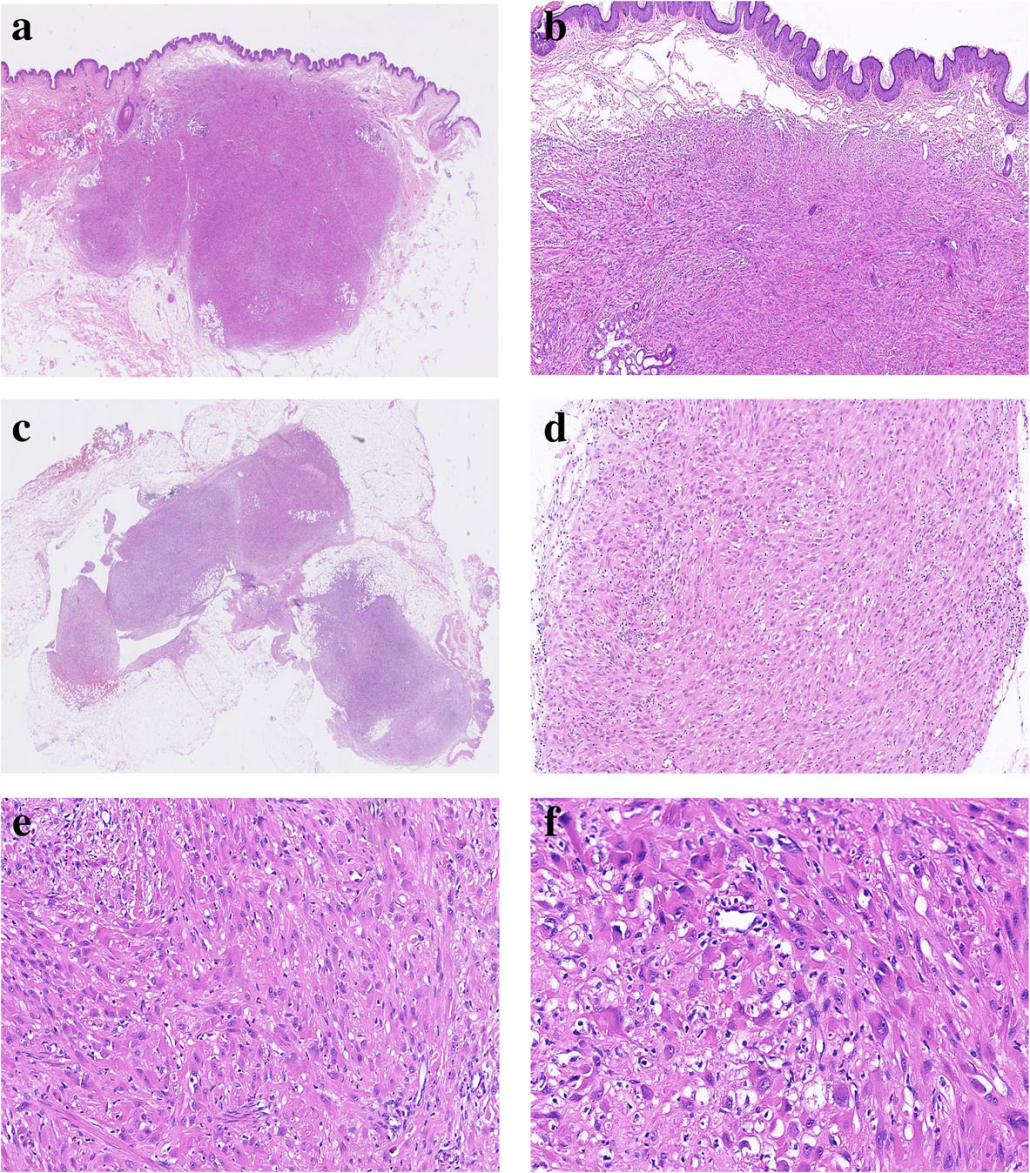
Scanning image of “mass of right labium majus (epidermis)” displayed a vague nodular appearance of the tumor (**a**), which was mainly dermal-based (**b**). Scanning image of “mass of right labium majus (deep)” showed a subcutaneously located tumor with multinodular architecture (**c**). The tumor was composed of fascicles of plump spindled to epithelioid cells with abundant eosinophilic cytoplasm (**d**), some of which resembled rhabdomyoblasts (**e**). Focal storiform arrangement was present and scattered neutrophilic infiltrate was observed in the stroma (**f**).

Microscopically, the “mass of right labium majus (epidermis)” was dermal-based (Fig. 1a, b), whereas the “mass of right labium majus (deep)” was located within the subcutaneous adipose tissue, assuming multinodular architecture (Fig. 1c). On high power, they were composed of fascicles or sheets of plump spindled to epithelioid cells with abundant eosinophilic cytoplasm, closely resembling rhabdomyoblasts (Figs. 1d, e). Focal storiform arrangement was also present (Figs. 1f). The tumor cells had oval to round vesicular nuclei with small nucleoli. Nuclear atypia is mild and mitotic activity was scarce (< 5/50 high power field). There was a prominent neutrophilic infiltration in the stroma. Coagulative necrosis was absent.

**Fig. 2:**
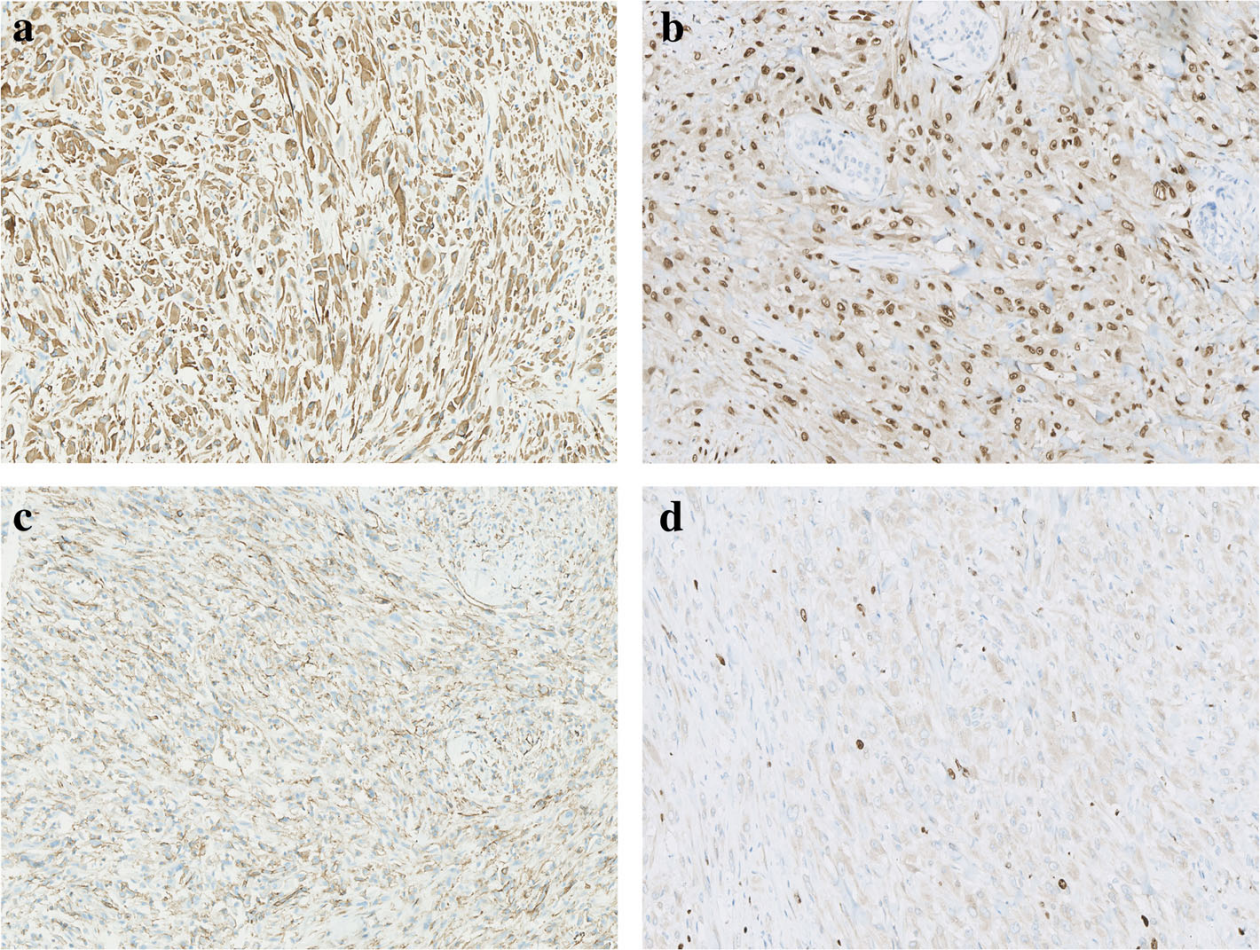
Tumor cells showed diffuse staining of AE1/AE3 (**a**) and ERG (**b**) with partial expression of CD31 (**c**). The Ki67 index was relatively low (**d**).

Immunochemically, tumor cells were diffusely positive for AE1/AE3, ERG and Fli1 (Fig. 2a, b), and partially positive for CD31 (Fig. 2c). The Ki67 index was about 2% (Fig. 2d). Intact staining of INI-1(SMARCB1) was retained. The tumor cells were negative for EMA, CD34, α-SMA, MSA, desmin, myogenin, MyoD1, calponin, ER and PR. The results of immunochemical study were summarized in Table 1.

**Table 1:** Immunohistochemical results of pseudomyogenic hemangioendothelioma

Antibody	Clone	Source	Dilution	Result
Pancytokeratin	AE1/AE3	Dako	1:100	+
EMA	E29	Dako	1:200	–
CD31	JC70A	Dako	1:80	+
CD34	QBend 10	Dako	1:50	–
ERG	EPR3864	Roche	Ready-to-use	+
Fli1	G146–222	BD Biosciences	1:100	+
α-SMA	1A4	Dako	1:400	–
MSA	HHF35	Dako	1:300	–
Desmin	D33	Dako	1:500	–
Calponin	CALP	Maixin China	1:150	–
Myogenin	MYF4	Novocastra	1:500	–
MyoD1	MYF3	Dako	1:50	–
SMARCB1(INI1)	25/BAF47	BD Biosciences	1:50	Retained
ER	PPG5/10	Dako	1:35	–
PR	PgR 636	Dako	1:50	–
Ki67	MIB1	Dako	1:150	2%

## Discussion and conclusion

The morphology and immunophenotype of the current case are in consistency with a typical PMHE, a distinctive vascular neoplasm which has been included in the 4th edition of WHO classification of Soft Tissue and Bone Tumors [5]. Although being well recognized as a novel entity of hemangioendothelioma, PMHE is known for lack of morphological evidence of endothelial differentiation. The descriptive term of ‘pseudomyogenic’ attributes to the neoplastic cells’ striking resemblance to rhabdomyoblasts in cytomorphology, which may lead to a misdiagnosis of a myogenic neoplasm, as illustrated in the current case as well as reported in the literature [6].

PMHE represents a rare neoplasm. Up to present, approximately 140 cases of PMHE have been reported in the English literature [7–9]. There was a male predominance with a gender ratio of 3.3:1. The majority occurred in young to middle aged adults with a mean and median age of 32 and 27 years respectively (range: 5 to 82 years). The peak age of incidence was in the second to fourth decades, which accounted for about 70% of all patients. Children (younger than 10 years) and elderly people (older than 60 years) were rarely involved.

Approximately 75% of PMHE arose in the skin and soft tissues, including those with concurrence of bone. Nearly one fourth of cases presented as primary bone lesions [10, 11]. With regard to site, over half of PMHE occurred in the lower extremities, including foot, leg, knee and thigh. About 17% occurred in the upper extremities, including forearm, hand, finger, upper arm, wrist and axilla; 15% in the trunk, including abdominal wall, chest wall, back and pelvis; 6% in the head and neck region, including face, oral cavity and neck; and exceedingly unusual site like the penis [12]. To date, occurrence of PMHE in the vulva has not been reported thus far. The current case is the first case of PMHE arising in the female external genitalia.

The clinical manifestation of PMHE was nonspecific. Most patients presented with multiple small painless or painful nodules which were rarely suspected as vascular lesions by clinicians. The clinical diagnosis embraced a wide variety of disease, including cutaneous cyst, sebaceous cyst, mosquito bite, abscess, nodular fasciitis, keratoacanthoma, spiradenoma, dermatofibroma, leiomyoma, neuroma, hemangioma and angiosarcoma [2, 7, 12–15]. In our case, the lesion was originally considered Bartholin gland cyst, vulvar hematoma or papilloma. It is worthy to note that patients with PMHE may present with a few visible cutaneous nodules which might not arise the clinicians’ alert. As about 70% of PMHE presented with multifocal disease which may involve multiple tissue planes in an anatomic region, it is essential for the patients to have further radiological examinations to make sure if there are occult intramuscular nodules or associated concurrence of bone lesions [2, 16].

On histological examination, our case displayed the characteristic features of PMHE that consisted of fascicles and sheets of plump spindle-to round shaped cells with vesicular nuclei, inconspicuous nucleoli and abundant homogeneous eosinophilic cytoplasm, mimicking rhabdomyoblasts. However, neoplastic cells were negative for myogenic markers such as desmin and myogenin. The epithelioid cytomorphology and immunoreactivity of AE1/AE3 might suggest an epithelioid sarcoma, one of SWI/SNF complex-deficient soft tissue neoplasms, which may also occur in the vulvar region. However, EMA and CD34 immunonegativity, and intact expression of INI1 in PMHE helped in the distinction from epithelioid sarcoma. Because of epithelioid morphology and endothelial differentiation, PMHE need to be differentiated from epithelioid vascular neoplasms, including epithelioid hemangioendothelioma, epithelioid hemangioma and epithelioid angiosarcoma. Epithelioid hemangioendothelioma (EHE) is characterized by cords and nests of epithelioid cells in a myxohyaline stroma. Tumor cells in EHE with *WWTR1-CAMTA1* fusion typically express CAMTA1 or show *CAMTA1* gene rearrangement by molecular analysis. A small percentage of EHE with *YAP1-TFE3* fusion express TFE3 or harbor *TFE3* gene rearrangement [17]. Epithelioid hemangioma is a benign vascular neoplasm composed of well-formed vessels lined by epithelioid endothelial cells with abundant eosinophilic cytoplasm and enlarged round nuclei. In contrast, PMHE lacks well-formed neoplastic vessels. Epithelioid angiosarcoma is a malignant vascular tumor composed of sheets of large, atypical epithelioid cells with vesicular nuclei containing prominent large central nucleoli, often showing focal vasoformation [18].

The endothelial nature of PMHE was usually demonstrated with the application of a panel of endothelial markers, including CD31, ERG and Fli1. Whereas ERG was consistently expressed in PMHE, the expression of CD31 was only seen in about 50~60% of PMHE. FOSB, a new marker derived from *SERPINE1-FOSB* fusion transcripts, was considered a highly sensitive in PMHE as diffuse nuclear immunoreactivity for FOSB (> 50% of cells) has been shown greater than 96% [19, 20]. As diffuse nuclear staining of FOSB was rarely observed in histologic mimics, FOSB was considered a diagnostic adjunct. However, what needs to be pointed out is that diffuse FOSB staining could be also observed in 54% of epithelioid hemangioma, and occasional cases of proliferative fasciitis, nodular fasciitis, and rarely epithelioid angiosarcoma, spindle cell angiosarcoma and epithelioid hemangioendothelioma [20, 21]. In addition, focal expression of FOSB has been demonstrated in a variety of lesions, including 50% of epithelioid sarcoma, 40% of epithelioid angiosarcoma, 55% of nodular fasciitis, and 40% of cellular fibrous histiocytoma [20]. Therefore, focal weak staining of FOSB was thought not diagnostic.

At molecular level, a balanced t (7;19)(q22;q13) has been found as the sole anomaly in PMHE [22]. Subsequent studies validated *SERPINE1-FOSB* gene fusion as the recurrent genetic alteration in PMHE [23]. Recently, a novel *ACTB-FOSB* gene fusion has been identified in PMHE by means of MSK-fusion solid assay [24]. It was found that tumors harboring *ACTB-FOSB* fusion more often presented with a solid pattern compared to those associated with *SERPINEI-FOSB* fusion [3]. FISH has been increasingly used in routine practice in the genetic analysis of translocation-related tumors. A precise diagnosis of PMHE can be also reached by using FISH analysis with split apart probes flanking *SERPINE1/ACTB* and *FOSB* genes, and further confirmed by RT-PCR assay [12].

Biologically, most of PMHE showed an indolent clinical course with a predilection for local recurrence, the rate of which was about 20% after a period ranging from 2 to 96 months after diagnosis [1, 2, 8]. Approximately one tenth of patients developed metastatic disease, including lung, regional lymph nodes, groin, subpleural region, ribs, and vertebrae [2, 11, 13, 25–29]. Few reports demonstrated that a PMHE could have a rapid progression and aggressive behavior [16, 30]. To date, only one case with numerous lytic bone lesions showed a spontaneous regression of the disease with PET/CT [27]. To avoid any risk of relapse, our patient had undergone a supplementary wide local excision and she remained well with no evidence of disease at last follow-up.

In the context of treatment, local resection is the mainstay for patients presenting with a localized disease. For patients with multifocal unresectable lesions or suffering metastatic disease, systemic chemotherapy is recommended. Most recently, target therapies such as mTOR inhibitors sirolimus, everolimus and rapamycin have been demonstrated to be effective treatment options in PMHE [28, 29]. It was supposed that treatment with the mTOR inhibitors resulted in a reduction in expression of the *SERPINE-FOSB* fusion protein. Similar inhibitory effect has been shown with telatinib treatment [31], an available *VEGFR1–4/PDGFRA *inhibitor which blocked *VEGF* signaling and down-regulated *SERPINE1*, affecting the self-regulation of the fusion gene. Target therapy provided a promising treatment option for patients with inoperable PMHE that failed with standard traditional chemotherapy.

In summary, we present a unique case of PMHE which occurred primarily in the vulva of a 51-year-old woman. Albeit exceedingly rare, PMHE should be included in the differential diagnostic list of vulvar tumors with spindled to epithelioid morphology and cytokeratin-positive immunophenotypes.

## Data Availability

Please contact author for data requests.

## Abbreviations

FISH: Fluorescenece in situ hybridization
PET/CT: Positron emission tomography/computed tomography
PMHE: Pseudomyogenic hemangioendothelioma
